# Fifteen years of the Diversity Outbred mouse model: a review

**DOI:** 10.1007/s00335-026-10196-6

**Published:** 2026-01-28

**Authors:** Zachary Tatom, Michael F. Miles, Abraham A. Palmer

**Affiliations:** 1 Department of Psychiatry, University of California San Diego, La Jolla, CA USA; 2 Department of Human and Molecular Genetics, Virginia Commonwealth University, Richmond, VA USA; 3 Alcohol Research Center, Virginia Commonwealth University, Richmond, VA USA; 4 Department of Pharmacology and Toxicology, Virginia Commonwealth University, Richmond, VA USA; 5 Department of Neurology, Virginia Commonwealth University, Richmond, VA USA; 6 Institute for Genomic Medicine, University of California San Diego, La Jolla, CA 92093 USA

## Abstract

Mouse models have played a critical role in complementing human genetics research due to their genetic similarity to humans and well-annotated and tractable genome. For over 15 years, the Diversity Outbred (DO) mice have existed as a powerful tool for mapping complex traits. With eight founder strains contributing to high levels of genetic diversity, heterozygosity, and large numbers of recombination events, DO mice allow for high-resolution genetic mapping. DO mice have been used to dissect the genetic architecture of physiological traits like blood lipids, behavioral traits such as cocaine self-administration, and molecular phenotypes such as gene expression across various tissues. Here we aim to exhaustively catalog DO mouse studies over the last 15 years, including both mapping and non-mapping studies, as well as to provide an overview of software tools and online resources related to the model.

**Supplementary Information:**

The online version contains supplementary material available at 10.1007/s00335-026-10196-6.

## Introduction

Animal models offer certain advantages over humans for human research. For example, animal models allow for control of environmental factors at a level that is not feasible in human subjects (Nieto et al. 2021; Vierkant et al. 2023). Use of these animal models can complement human genetics research of complex traits by either using reverse genetics approaches to validate human GWAS candidate genes from human GWAS in the model organism or by using forwards genetics approaches to map phenotypes in the model organism directly (Lathen et al. 2020). Animal models also allow for follow-up using experimental manipulations that are not possible or not ethical in human subjects, such as the use of CRISPR/Cas9 technologies (Tamura and Toda 2020). Mice are one of the most ubiquitous model organisms in mammalian genetics research due to their relatively easy and low-cost laboratory maintenance, the availability of inbred strains, and ability to routinely carry out genetic manipulation (Long et al. 2022). Mice are also genetically similar to humans, with about 40% of the nucleotide sequence of humans able to be aligned to the mouse genome and 80% of mouse genes having a single ortholog in humans (Mouse Genome Sequencing et al. 2002).

**Fig. 1:**
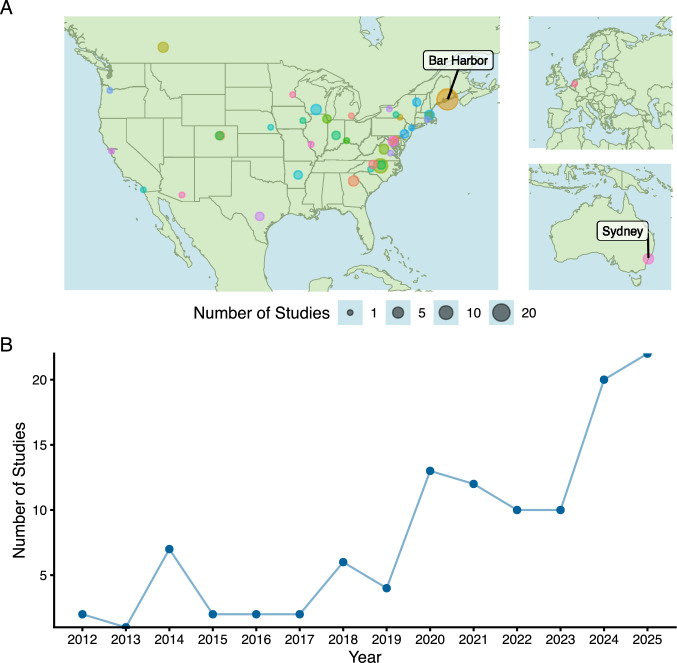
Diversity Outbred mouse publications by location and over time. We identified 112 DO and DOz mouse studies published since the first paper about the model in 2012. Most of these were in North America, where the DO colony is maintained at the Jackson Laboratory in Bar Harbor, Maine; the DOz colony is maintained at the University of Sydney in Sydney, Australia (**A**). 2025 has shown the largest number of published DO and DOz mouse studies to date with 22, followed by 2024 with 20 (**B**)

Two of the most powerful mouse models for genetic studies are the Collaborative Cross (CC) mouse and the Diversity Outbred (DO), both derived from the same eight founder strains. These founders include five laboratory strains (129S1/SvImJ, A/J, C57BL/6J, NOD/ShiLtJ, and NZO/HlLtJ) and three wild-derived strains, each derived from distinct subspecies (CAST/EiJ derived from the Southeast Asian *Mus musculus castaneus*, PWK/PhJ derived from the European *Mus musculus musculus*, and WSB/EiJ derived from the North American *Mus musculus domesticus*) (Churchill et al. 2004). While recombinant inbred lines created from only two parent strains (like BXD mice) are limited in the genetic variation to only alleles originating in those two strains, the CC founders were initially selected to capture an estimated 89% of the total variation in the mouse genome (Roberts et al. 2007). The CC was designed with a goal of developing 1000 inbred strains; however, breeding difficulties have led to far fewer lines being available, with 63 currently available from the colony maintained at UNC Chapel Hill. In part to compensate for the challenges in establishing inbred lines, as well as to have an outbred resource, the DO mouse population was established in 2009 by incorporating a random mating design across 144 partially-inbred CC lines (Churchill et al. 2012), with an independent colony developed from 29 breeding pairs spanning 55 CC strains later established in Australia (known as DOz) (Masson et al. 2023) (Fig. 1A).

The DO mouse population has been used to dissect the genetic architecture of complex traits including physiological, behavioral, and molecular phenotypes. The model continues to see increased adoption with 22 DO mouse studies published in 2025, the most in any single year to date (Fig. 1B). We have identified 112 DO studies published at the time of writing by conducting a PubMed search for “Diversity Outbred Mouse”, manually collecting sample size and phenotypes measured from manuscript text (Table 1). By our estimation, over 30,000 DO mice have been phenotyped for published studies so far, with about 80% (~ 24,000) of those having also been genotyped (Table 1). While a few studies have used this population without genotyping—usually to determine if genetic background may be contributing to a trait prior to further study—the genetic diversity of the DO mouse represents a novel complement to human genome-wide association studies (GWAS) and a powerful tool for genetic mapping on its own.

**Table 1:** Survey of Diversity Outbred mouse studies from 2012–2024

Publication	Sample size	Traits measured
Al-Barghouthi et al. (2021)	150	Lipids, glucose, insulin, body weight, percent fat, lean tissue mass
Svenson et al. (2012)	150	Lipids, glucose, insulin, body weight, percent fat, lean tissue mass
Logan et al. (2013)	283	Open field, light–dark box, visual cliff avoidance, tail suspension
Recla et al. (2014)	283	Hot plate
Munger et al. (2014)	277	Liver RNA-seq, lipids, glucose, insulin, body weight, percent fat, lean tissue mass
Gatti et al. (2014)	5284	Neutrophil counts
Smallwood et al. (2014)	292	Plasma cholesterol, glucose, triglycerides, insulin; atherosclerotic lesions; Apobec1 mRNA; ApoB protein
Koh et al. (2014)	171	Spatial memory in water maze; hippocampal somatostatin expression
Church et al. (2015)	272	Liver necrosis, serum alanine aminotransferase
Dickson et al. (2015)	98	Open field, novelty preference in divided open field, exploration in hole board apparatus, cocaine IVSA
French et al. (2015)	600	DNA damage via micronucleus frequency in reticulocytes and mature erythrocytes
Niazi et al. (2015)	166	Immune cytokines and chemokynes from blood and lung
Gu et al. (2016)	277	Liver RNA-seq, RNA editing cite prediction lipids, glucose, insulin, body weight, percent fat, lean tissue mass
Chick et al. (2016)	192	Liver proteomics, liver RNA-seq
Winter et al. (2017)	493	Prostate weight, para-aortic lymph node weight, lung and liver lesion count, prostate RNA-seq
Percival et al. (2018)	1036	Micro-computed tomography of heads: zygomatic arch length, saggital cranial vault length, midline cranial base length
Harrill et al. (2018)	90	Kidney histology, blood chemistry (sodium, potassium, chloride, total carbon dioxide, ionized calcium, glucose, urea nitrogen (BUN), creatinine (sCre), hematocrit, hemoglobin, and anion gap), urinary proteins, urine RNA microarray
Shorter et al. (2018)	609	Heart weight, body weight, femur length
Gatti et al. (2018)	972	Body weight, blood cell counts
Mayeux et al. (2018)	280	Silica-induced autoimmunity markers, body weight, spleen weight
Yuan et al. (2018)	502	Body weight, testis weight
Coffey et al. (2019)	288	Plasma choline metabolites, RNA microarrays
Keller et al. (2018)	483	Body weight; plasma insulin, blood, and triglycerides; oral glucose tolerance;
Recla et al. (2019)	300	Formalin assay of nociception
Kemis et al. (2019)	500	Plasma bile acid, cecal bile acid, body weight, bacterial taxa
Yang et al. (2019)	270	Pancreatic cancer lesions, body weight
Kurtz et al. (2020)	30–160	Tuberculosis organ burdens following vaccination or control; body weight
Keenan et al. (2020)	338	Sleep cycle, sleep latency, voluntary wheel running
You et al. (2020)	450	Body weight, alkaline phosphatase, alanine aminotransferase, aspartate aminotransferase, total bilirubin, blood urea nitrogen, liver triglycerides, liver RNA-seq
Katz et al. (2020)	1147	Body mass, facial allometry
Huda et al. (2020)	120	Plasma and urine cystatin C
Pal et al. (2020)	19	Body mass, fat mass; fasting glucose and fasting insulin before and after treatment
Que et al. (2021)	292	Liver RNA-seq
Ouellette et al. (2020)	489	Spontaneous continuous alternation T-maze
Skelly et al. (2020)	183	Gene expression and chromatin accessibility in mouse embryonic stem cells
Bubier et al. (2020)	300	Breath rate, recovery, and survival time following morphine administration
Wei et al. (2020)	84	Her2-binding Ab in immune serum
Linke et al. (2020)	384	Plasma lipidomics
Patterson et al. (2020)	513	Body weight, radiation exposure dose–response relationship curve
Tavolara et al. (2020)	452	Tuberculosis susceptibility (via lung imaging/ML)
Kim et al. (2021)	329	Skin fibroblast cellular rhythm
Toussaint et al. (2021)	98	Head shape / facial allometry
Tavolara et al. (2021)	77	Microarray gene expression / predicted gene expression
Harper et al. (2021)	57	Retinal ganlia response to blast-mediated traumatic brain injury
Al-Barghouthi et al. (2021)	619	Body weight, body length, skeletal phenotypes
Takemon et al. (2021)	200	Kidney function (GFR), urinary albumin, creatinine, kidney RNA-seq
Koyuncu et al. (2021)	657	Body condition, respiratory function, panel of protein biomarker candidates for tuberculosis
Gould et al. (2021a, b)	348	Serum blood urea nitrogen, renal glutathione
Gould et al. (2021a, b)	351	Body weight, liver weight, hepatic glutathione
Starcher et al. (2021)	225	Body weight, heart weight, serum GDF11, circulating myostatin level
Keenan et al. (2021)	325	Sleep cycle, EEG, EMG
Zhang et al. (2022)	960	Grip strength, body metrics (length, weight, fat, lean mass, etc.), rotarod, acoustic startle, wheel running, echo-cardiogram
Parker et al. (2022)	798	Ethanol-induced ataxia, hypothermia, and loss of righting reflex
Al‐Shaer et al. (2022)	250	Body weight, fat mass distribution, serum total gastric inhibitory polypeptide, glucagon, insulin, glucose, leptin, and resistin
Wright et al. (2022)	960	Body weight over lifetime
Percival et al. (2022(	1071	Neurocranial / endocast estimation, tibia length
Ouellette et al. (2022)	960	Y-maze working memory, contextual fear memory
Moran et al. (2022)	47	Intramembranous bone regeneration, skeletal traits
Xiao et al. (2022)	163	Brown adipose tissue proteomics
Xenakis et al. (2022)	75	Body weight, body composition, blood glucose, plasma insulin, before and after inorganic arsenic exposure
Bagley et al. (2022)	525	Reversal learning, open field, light–dark box, hole board, novel place preference
Chen et al. (2022)	406	Physiological aging and resilience
Gerdes Gyuricza et al. (2022)	185	Transcriptomic and proteomic data in whole-heart aging
Chapp et al. (2023)	26	Locomotor activity following cocaine sensitization
Binh Tran et al. (2023)	228	Intravenous cocaine self-administration, fecal microbiome measurements
Aydin et al. (2023)	190	Gene expression and chromatin accessibility in mouse embryonic stem cells
Ruby et al. (2023)	228	Frailty index of aging
Chella Krishnan et al. (2023), Ruby et al. (2023)	~ 500	Heart, islet, skeletal muscle, adipose, and liver expression; heart weight
Kim et al. (2023)	461	Plasma lipids and glucose, aortic plaque size
Masson et al. (2023)	250	Fat and lean mass, body weight, glucose tolerance, blood glucose and insulin, skeletal muscle proteomics
Philip et al. (2023)	386	Striatum RNA-seq
Dillard et al. (2023)	5	Bone marrow stromal cell scRNA-seq
Price et al. (2023)	500	Plasma lipoprotein size, lipoprotein subclass
Cruz Cisneros et al. (2024)	126	Weight, SARS-CoV-2 viral titer, serum antibody
Specht et al. (2024)	20	IgG reaction to SARS-CoV-2 spike protein
Cousineau et al. (2024)	840	Weight, BMI, fat mass, cholesterol, calcium
Rasquinha et al. (2024)	88	Serum, heart, pancreas histology following mt10 vaccination
Kurtz et al. (2024)	~ 1100	Tuberculosis bacterial burden
O’Connor et al. (2024)	32	Fibroblast morphology
Kuffler et al. (2024)	176	Genetic-epigenetic interactions
Migotsky et al. (2024)	50	Bone mineral content, fasting blood glucose, DEXA scans, bone morphology, osteocyte morphology, cortical bone traits
Patil et al. (2024)	30	Response to ocular herpes infection
Gatti et al. (2024)	850	Weigh, lung granuloma necrosis, tuberculosis lung burden, lung cytokines and chemokines
Nemkov et al. (2024a), Nemkov et al. (2024b)	525	Carnitine pools in red blood cells
Oliveira et al. (2024)	189	Dendritic cell counts in lymphoid and non-lympphoid tissues
Koyuncu et al. (2024)	1009	Tuberculosis lung burden, lung histology, lung gene expression
Thillainadesan et al. (2024)	62	Dietary energy intake, body weight, body composition, glucose, insulin, metabolic assays in plasma, liver, adipose tissue
Koch et al. (2024)	346	Liver histology score, renal and hepatic glutathione redox markers
Van Megen et al. (2024)	549	Age-related urinary magnesium excretion
Nemkov et al. (2024a), Nemkov et al. (2024b)	525	Red blood cell spontaneous storage hemolysis test
Mignogna et al. (2024)	636	Voluntary ethanol consumption, light–dark box, marble burying, prefrontal cortex and nucleus accumbens bulk RNA-seq
Luciano et al. (2024)	246	Frailty index of aging
Plett et al. (2024)	29	Lifespan in Hematopoietic Syndrome of Acute Radiation Syndrome survivors
Reisz et al. (2024)	525	Metabolite quantitative trait loci
D’Alessandro et al. (2025)	350	Metabolites and lipids in red blood cells
Sweet et al. (2025)	50	Body composition
Lien et al. (2024)	14	Glucose and lipid metabolism
Williams et al. (2025)	~ 36	Airway hyperinnervation and hyperresponsiveness after high-fat diet
Faizan et al. (2025)	~ 7–9	Inflammatory response to acute exposure to cigarette smoke
Aydin et al. (2025)	127	eQTL across two cell types (mouse embryonic stem cell and neural progenitor cell)
Parikh et al. (2025)	85	Metyhlation in stem cells
Sabnis et al. (2025)	313	Frailty assessment using machine vision
Paules et al. (2025)	300	Body compostion, blood glucose, and plasma hormone levels following calorie restriction
Mullis et al. (2025)	2444	Longevity
Aydin et al. (2025)	127	Expression in neural progenitor cells
Kurtz et al. (2025)	57	Response to tuberculosis challenge following vaccination
Finch et al. (2025)	11	Hippocampal neurogenesis
Widmayer et al. (2025)	49	Comparison of genome sequencing vs. genotyping by array for haplotype reconstruction
Tyler et al. (2025)	482	Mediation of eQTLs on metabolic traits
Madsen et al. (2025)	399	Muscle lipids and insulin resistance
Kavushansky et al. (2025)	309	Behavioral outcomes following early-life stress
Masson et al. (2025)	670	Glucose homeostasis
Dickson et al. (2025)	65	Cocaine self-administration
Fu et al. (2025)	835	Body composition, body weight, clinical chemistry, electrocardiogram, organ weight, and urine chemistry
Keele et al. (2025)	350	Protein, metabolite, and lipid QTL from red blood cells

112 studies were identified as published during this time period using PubMed Search for the phrase “Diversity Outbred Mouse.” Sample sizes and traits measured were identified from individual manuscripts.

### Genetic mapping in DO mice

Resolution in QTL mapping is a function of the number of recombination events between parental strains because association analysis relies on recombination events to map traits and reduced linkage disequilibrium between neighboring markers allows for sharper localization (Valdar et al. 2006). F2 populations generated by recombinant inbred lines (RIL), such as the BXD mouse, are able to reach genetic mapping on the order of 6–20 Mbp (Ashbrook et al. 2021; Fossey et al. 2001) and often require follow-up fine-mapping studies using alternative models to map more precise loci (Demarest et al. 2001).

Unlike the CC and RIL such as the BXD, each DO mouse is genetically unique, with a high degree of heterozygosity (Churchill et al. 2012) as seen in humans. It has been estimated that the outbred mating scheme doubles the effective population size while minimizing selection of allele frequencies, enabling estimation of additive genetic effects using a possible 36 diplotype states (Churchill et al. 2012; Rockman and Kruglyak 2008). A sample size of 640 mice has been estimated as sufficient to map a private allele (frequency 1/8) with a recessive effect that shifts a trait by 1 mean standard deviation at adequate power (*α* = 0.05, 1—*β* = 0.80) (Churchill et al. 2012), allowing for genetic mapping up to a sub-Mbp resolution (Churchill et al. 2012; Rockman and Kruglyak 2008). As additional generations of DO mice are bred, further recombinations will occur which are expected to increase mapping resolution.

### Tools available for DO mouse research

Methods for genetic mapping studies in DO mice typically include linkage-based quantitative trait locus (QTL) mapping using R/QTL2 (Broman et al. 2019) [or in older studies, DOQTL (Gatti et al. 2014)] software [although one study used a GWAS approach (Yang et al. 2019)]. R/QTL2 also allows for haplotype reconstruction and analysis, estimating contributions of the eight founder strains to an observed phenotype (Broman 2022). The Jackson Laboratory has also developed software for reconstructing genotypes from RNA-seq data, allowing for more sensitive alignment of RNA-seq reads and identification of allele-specific expression (Choi et al. 2020; Mignogna et al. 2024).

Online resources available for depositing and accessing DO mouse data include the Mouse Phenome Database (https://phenome.jax.org/) for phenotype data (Bogue et al. 2015), GeneNetwork (https://genenetwork.org/) for mapping results in both DOs and various other inbred and outbred species (Mulligan et al. 2017), GeneWeaver (https://www.geneweaver.org/) for gene sets resulting from studies (Baker et al. 2012), and the Diversity Outbred Database (https://divdb.jax.org/) as a genotype data repository from JAX. The QTLViewer (https://qtlviewer.jax.org/) provides both a point-and-click interface for R/QTL2 and a tool to download R/QTL2-formatted data from DO Mouse studies. QTLViewer allows for both local instances to be set up by individual researchers or for users to submit data to JAX’s collection (Vincent et al. 2022). However, there is no utilization across DO mouse studies as to a single repository for these data, with many researchers uploading data to Figshare, Dryad, or other online resources.

### Challenges in DO mouse research

Because individual DO mice are genetically distinct, they must each be genotyped on an individual basis, unlike with inbred lines. While the third-generation Mouse Universal Genotyping Array (i.e., GigaMUGA) platform provided by Neogen has been optimized for the CC and DO mouse populations (Morgan et al. 2015), scaling the use of this array to a sample large enough to detect loci of low effect size in genetic mapping remains somewhat expensive, with each array costing $103 (at the time of this writing). However, an alternative to GigaMUGA is low-coverage whole-genome sequencing which may reduce cost up to 50%, capture de novo mutations, provide better coverage, and potentially more accurately model haplotypes (Widmayer et al. 2025).

Additionally, it is not possible to assay invasive phenotypes multiple times on the same genetic background—for example, to take brain tissue for gene expression both before and after long-term exposure to a drug. Replicating genotypic findings in smaller samples of DO mice can also be difficult due to the increased heterogeneity, and studies requiring pre- and post-exposure measurements require separate cohorts of animals. A number of studies have also used cell lines which provide he opportunity for replication by using cells from a single individual for repeated measurements either for replication or to explore how different environmental conditions effect specific genotypes. (Aydin et al. 2023; O’Connor 2024; Skelly et al. 2020) This method is well-suited to molecular phenotypes such as gene expression but is not appropriate for behavioral traits or some physiological measures. Other mouse models, such as the CC or BXD, do not share these genotype replicability challenges and may be more appropriate for certain applications. The extensive expansion of BXD lines in particular offers high resolution mapping and an extensive history of phenotypic data but suffers from lower genetic diversity due to use of only 2 parental lines (Ashbrook et al. 2021). CC lines offer the possibility of genetic replicability using the same 8 founders as the DO mouse, with F1 crosses (i.e., CC-recombinant intercross or CC-RIX) allowing for this replicability across this genetic background (Graham et al. 2015). Researchers should carefully consider which mouse model to use based on whether their study design requires genotypic replicability.

Lastly, despite efforts to design a breeding scheme that minimizes allele selection there have been disruptions to allele balance, most notably a locus on mouse chromosome 2 which previously was found to be enriched for alleles from one founder strain, WSB/EiJ. This region was found to affect sperm function, with WSB/EiJ alleles providing a selective advantage at this locus. These alleles have since been removed from the breeding colony. As a result, there is a loss of private WSB/EiJ alleles for this region on chromosome 2 and a disruption to the balance of the other 7 founder alleles at this locus, somewhat complicating genetic analyses for the chromosome (Chesler et al. 2016).

## Conclusions

The DO mouse is a powerful tool for genetic research, particularly in studies of complex traits where genetic diversity and phenotypic variance are important. The population’s high degree of heterozygosity and large number of recombination events allow for the mapping of genetic variants with finer resolution than other mouse models, complementing human genetics research. However, the inherent genetic variability in DO mice presents challenges, such as increased costs for genotyping and difficulty in replicating genotypes. Nevertheless, a growing number of software tools and online resources, ever-increasing generation numbers leading to higher mapping resolution, and expanding adoption of the model across diverse phenotypes all demonstrate the utility of the DO population in the broader context of genetics research.

## Supplementary Information

Below is the link to the electronic supplementary material.

Supplementary file1 (XLSX 15 kb)

## Data Availability

No datasets were generated or analysed during the current study.
